# Family-based whole exome sequencing of atopic dermatitis complicated with cataracts

**DOI:** 10.18632/oncotarget.19739

**Published:** 2017-07-31

**Authors:** Wenxin Luo, Wangdong Xu, Lin Xia, Dan Xie, Lin Wang, Zaipei Guo, Yue Cheng, Yi Liu, Weimin Li

**Affiliations:** ^1^ Department of Respiratory and Critical Care Medicine, West China Hospital, Sichuan University, Chengdu, Sichuan, 610041, PR China; ^2^ Department of Rheumatology and Immunology, West China Hospital, Sichuan University, Chengdu, Sichuan, 610041, PR China; ^3^ State Key Laboratory of Biotherapy and Collaborative Innovation Center for Biotherapy, West China Hospital, Sichuan University, Chengdu, Sichuan, 610041, PR China; ^4^ Department of Ophthalmology, West China Hospital, Sichuan University, Chengdu, Sichuan, 610041, PR China; ^5^ Department of Dermatology, West China Hospital, Sichuan University, Chengdu, Sichuan, 610041, PR China

**Keywords:** atopic dermatitis, cataracts, mutation

## Abstract

**Background:**

Atopic dermatitis (AD) is a common skin disorder with elevated prevalence. Cataract induced by AD rarely occurs in adolescent and young adult patients, which is also called atopic cataract. Using whole exome sequencing, we aimed to explore genetic alterations among AD and atopic cataract.

**Result:**

We recruited a 19 year-old Chinese male with AD accompanied with cataracts, his father with AD and his mother without AD or cataract. Through analysis of the exomic sequence of the 3 individuals from the same family, we identified that with respect to AD, there were 162 genes mutated in both this patient and his father but not in his mother. In addition, we found 10 genes mutated in this patient only without in his parents according to cataract.

**Conclusion:**

This research suggests that coinheritance of mutations in these genes may correlate with AD, and the pathogenesis of AD complicated with cataracts was related to genetic factors.

## INTRODUCTION

Atopic dermatitis (AD) is a chronic, relapsing inflammatory skin disease with a worldwide prevalence of 8.7-18.1% in children [1] and 1.5-10.2% in adults [2]. It is characterized by continual itchiness, flares and sleep disturbance, negatively regulating the occupational activities and social relationships of patients, the quality of life of patients and their families [3]. Studies have convinced of a combination of genetic and environmental factors in the pathogenesis of AD. Genetic evidence depicts a complex network comprising epidermal barrier dysfunctions and dysregulation of innate and adaptive immunity in this disease. It has been accepted that mutations in the human filaggrin (*FLG*) gene are the most significant and well-replicated genetic mutations related to AD. Some other mutations such as *SPINK5*, *SPRR3*, and *CLDN1* may also correlate with epidermal barriers linked to AD. Genetic variants are able to contribute to the abnormal innate and adaptive responses, such as mutations in IL-1 family cytokines and receptors genes, vitamin D pathway genes, Th2 cytokines genes [4]. A cataract is a clouding of the lens that reduces light transmission to the retina, and it decreases the visual acuity of the bearer. It is one of the severe ocular complications of AD manifested in the eyes. The general classification of cataract includes nuclear, cortical and posterior subcapsular subtypes. Here, we focused on a Chinese male with AD complicated with cataracts via the recently developed whole exome sequencing approach, which has been used to determine the genetic basis of rare diseases.

## RESULTS

### Clinical description

**Figure 1:**
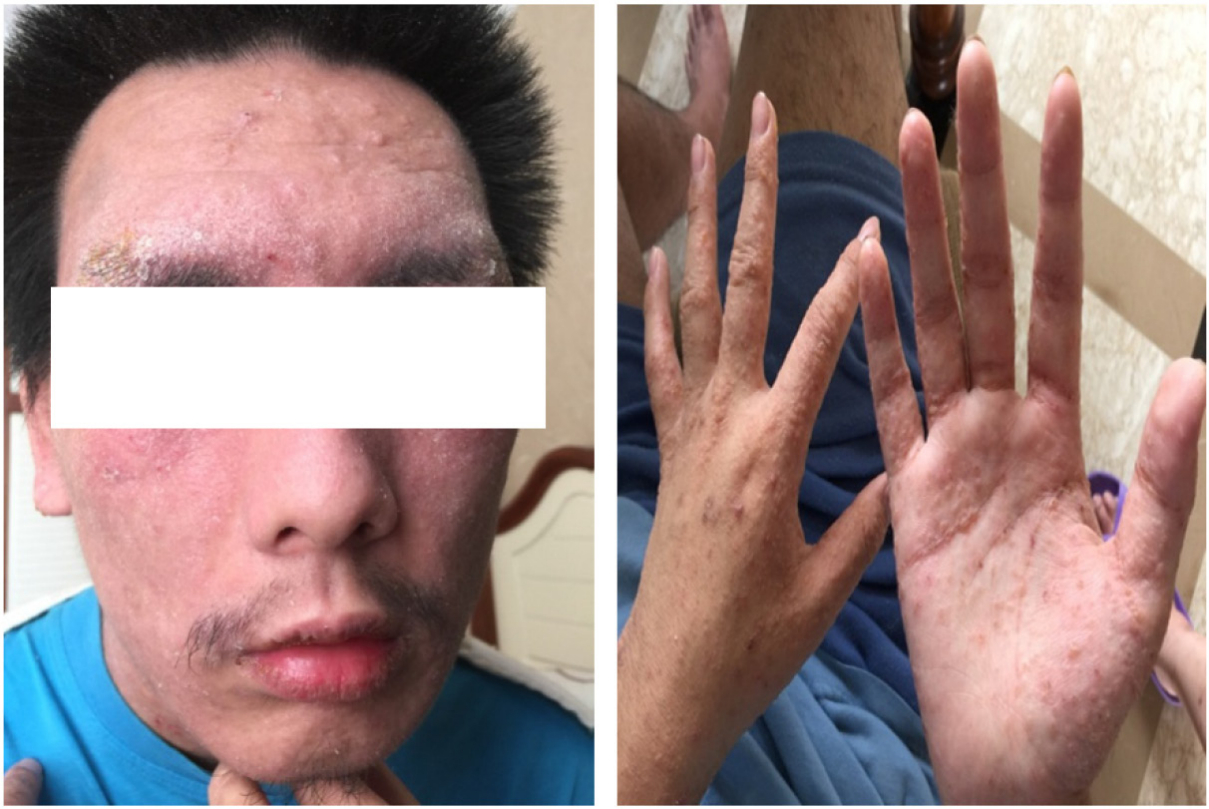
Appearance of red papules and scratches in the young patient

**Figure 2:**
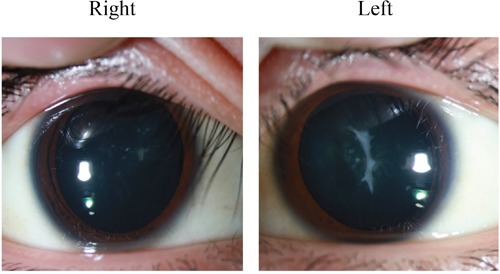
Anterior subcapsular cataracts and posterior subcapsular cataracts in both lens

**Figure 3:**
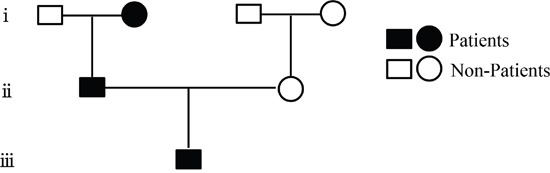
Family pedigree of the atopic dermatitis

The patient was a 19 year-old Chinese male who was admitted to our hospital with the chief complaint of relapsing generalized skin rash and blurred vision in August, 2015. His rash began from 10 years ago, accompanied with diffuse red papules all over the body, white desquamation, and skin itchiness. He was diagnosed with AD, and treatment without corticosteroids was not effective. There were persistent skin lesions, with obvious itchiness. His skin became dry and flaking, and some area became hard and thick. Seven months ago, his binocular vision became gradually declined. When admitted, red papules and scratches were displayed on the face, neck, trunk, and four extremities, especially on the face and neck (Figure 1). Both eyelids were hard and thick. The right vision was 0.4, and the left vision was 0.1. Keratic precipitates were negative, but both lens were turbid (Figure 2), of which the right one was more severe than the left. Hemogram analysis revealed eosinophile granulocyte 0.8×10^9^/l (12.1% in WBC), and immunological studies showed that expression of IgE was strongly elevated (>3000.00IU/ml). Other investigations such as expression of complements, immune complex, subpopulation of T cells, anti-double stranded DNA antibody, anti-nuclear antibody were normal. Serum allergen test indicated that combination of willow/poplar/elm, crab, shrimp, combination of dermatophagoides pteronyssinus/dermatophagoides culinae were positive allergens. He has a history of eczema and house dust allergy. Interestingly, his father and grandma were diagnosed with AD, respectively (Figure 3).

### Genetic analysis

Due to the rarity of cataract occurred in this young male with AD, we hypothesized that an underlying genetic alteration might be present in this patient. We discussed the genetic relationship between the patient and his parents by whole exome sequencing. In order to discover the candidate mutations of AD, we searched for the genes both mutated in this patient and his father but not in his mother. Results showed that 162 genes were both mutated in this patient and his father but not in his mother (Table 1, Supplementary Table 1).

**Table 1:** List of 162 genes both mutated in the patient and his father by whole exome sequencing

Chromosome	Position	Gene	SNP		Chromosome	Position	Gene	SNP	
chr1	93646190	*TMED5*	rs185712821	C/T	chr10	75563726	*NDST2*	NA	C/T
chr1	45271238	*PLK3*	rs55654497	G/A	chr11	5537592	*UBQLNL*	rs142657773	G/C
chr1	162569107	*UAP1*	rs190156359	T/A	chr11	74915493	*SLCO2B1*	rs192050675	C/A
chr1	214537946	*PTPN14*	rs200340171	G/A	chr11	78369215	*TENM4*	rs185503085	C/T
chr1	109260438	*FNDC7*	NA	T/C	chr11	123988461	*VWA5A*	rs202202178	A/T
chr1	158533225	*OR6P1*	NA	C/T	chr11	124266877	*OR8B3*	rs183842912	A/C
chr1	16907303	*NBPF1*	rs681623	C/T	chr11	10585620	*LYVE1*	NA	G/T
chr1	17083872	*MST1L*	rs11545933	G/A	chr11	118376389	*KMT2A*	NA	C/T
chr1	17263277	*CROCC*	rs200228265	G/A	chr11	130060375	*ST14*	NA	C/T
chr1	181019227	*MR1*	NA	G/A	chr11	3726498	*NUP98*	NA	G/A
chr1	232561368	*SIPA1L2*	NA	C/A	chr11	6661388	*DCHS1*	rs147698268	G/A
chr1	23637401	*HNRNPR*	NA	C/T	chr11	71249529	*KRTAP5-8*	rs200162819	G/A
chr1	248813827	*OR2T27*	rs1782241	T/C	chr12	6669359	*NOP2*	rs142370738	G/C
chr1	33237103	*KIAA1522*	NA	C/T	chr12	7475081	*ACSM4*	rs7485773	C/T
chr1	57411713	*C8B*	NA	G/C	chr12	105464439	*ALDH1L2*	rs199841702	G/C
chr1	86355260	*COL24A1*	NA	C/G	chr12	109217071	*SSH1*	rs140582047	T/A
chr1	9780232	*PIK3CD*	NA	G/A	chr12	110221524	*TRPV4*	rs55728855	C/T
chr2	90249249	*IGKV1D-43*	NA	T/C	chr12	112150408	*ACAD10*	rs145407775	C/T
chr2	113343610	*CHCHD5*	rs199612227	A/G	chr12	124097777	*DDX55*	rs117200049	G/A
chr2	209108226	*IDH1*	rs186787509	T/C	chr12	108956430	*ISCU*	NA	G/C
chr2	233735070	*C2orf82*	rs200597442	C/G	chr12	11183661	*TAS2R31*	NA	C/A
chr2	152484095	*NEB*	NA	C/G	chr12	12966365	*DDX47*	NA	G/A
chr2	179466289	*TTN*	NA	C/T	chr12	48104624	*ENDOU*	NA	C/T
chr2	187627500	*FAM171B*	NA	A/G	chr12	52885339	*KRT6A*	rs199613662	C/T
chr2	233675986	*GIGYF2*	NA	A/G	chr12	6950473	*GNB3*	NA	C/T
chr2	73315216	*RAB11FIP5*	NA	A/T	chr13	103419820	*TEX30*	rs200314758	T/C
chr2	97877478	*ANKRD36*	rs10194525	G/A	chr13	96242562	*DZIP1*	NA	T/G
chr3	7728055	*GRM7*	rs182447901	C/T	chr14	45432003	*FAM179B*	rs200775208	C/T
chr3	33644578	*CLASP2*	rs117166070	C/T	chr14	68241828	*ZFYVE26*	rs193244014	G/C
chr3	49751251	*RNF123*	rs117758999	G/A	chr14	105415264	*AHNAK2*	rs201041268	G/A
chr3	112648174	*CD200R1*	rs188572017	A/T	chr14	32256995	*NUBPL*	NA	G/A
chr3	151107788	*MED12L*	rs199780529	T/C	chr14	70925106	*ADAM21*	NA	T/C
chr3	124351317	*KALRN*	NA	G/A	chr15	45456025	*DUOX1*	rs186783799	G/A
chr3	132319977	*CCRL1*	NA	G/A	chr15	89402346	*ACAN*	rs188663484	T/C
chr3	40442466	*ENTPD3*	rs140869368	G/A	chr16	21994499	*UQCRC2*	NA	T/A
chr4	42119545	*BEND4*	rs187366202	G/T	chr16	15761154	*NDE1*	rs147283674	C/T
chr4	47788868	*CORIN*	rs186748019	C/A	chr16	55530864	*MMP2*	rs28730814	G/A
chr4	52948557	*SPATA18*	rs184617860	C/T	chr16	18849442	*SMG1*	NA	G/A
chr4	186291928	*LRP2BP*	NA	C/T	chr16	2287576	*DNASE1L2*	NA	C/T
chr4	4190576	*OTOP1*	rs2215642	C/G	chr16	28846489	*ATXN2L*	NA	T/C
chr5	38451559	*EGFLAM*	rs140968262	A/G	chr16	30100451	*TBX6*	rs202193096	G/A
chr5	94814011	*TTC37*	rs143227096	C/A	chr16	456349	*DECR2*	NA	C/T
chr5	137722246	*KDM3B*	rs184734460	C/G	chr16	46637519	*SHCBP1*	NA	A/G
chr5	178507048	*ZNF354C*	rs116562180	C/G	chr16	67991689	*SLC12A4*	NA	G/A
chr5	128442753	*ISOC1*	NA	G/T	chr16	71163611	*HYDIN*	NA	T/G
chr5	149357850	*SLC26A2*	NA	G/T	chr16	71961625	*IST1*	NA	C/G
chr5	171341357	*FBXW11*	NA	G/T	chr16	84213027	*TAF1C*	NA	C/G
chr6	26056145	*HIST1H1C*	rs79483116	G/A	chr17	76166705	*SYNGR2*	NA	G/A
chr6	27277365	*POM121L2*	rs61736085	G/A	chr17	36719794	*SRCIN1*	rs118094989	C/A
chr6	39847207	*DAAM2*	rs139876341	A/G	chr17	40714796	*COASY*	rs200009135	G/C
chr6	43017728	*CUL7*	rs146808129	C/A	chr17	48916935	*WFIKKN2*	rs35300894	G/A
chr6	83838955	*DOPEY1*	rs188246058	A/C	chr17	55918596	*MRPS23*	rs117734846	C/T
chr6	160485490	*IGF2R*	rs8191859	G/A	chr17	73096776	*SLC16A5*	rs116126425	G/A
chr6	119628121	*MAN1A1*	NA	C/T	chr17	11461158	*SHISA6*	NA	A/G
chr6	143825320	*FUCA2*	NA	A/G	chr17	12920199	*ELAC2*	rs140665334	G/A
chr6	34512160	*SPDEF*	rs375427681	G/A	chr17	14139300	*CDRT15*	rs11867613	A/G
chr6	34802049	*UHRF1BP1*	rs368713702	A/G	chr17	14204942	*HS3ST3B1*	NA	T/C
chr6	39893446	*MOCS1*	rs377167949	G/A	chr17	26823582	*SLC13A2*	NA	G/A
chr7	75617513	*TMEM120A*	rs372363121	C/T	chr17	2966032	*OR1D5*	rs2676564	C/G
chr7	141464509	*TAS2R3*	NA	T/C	chr17	5036211	*USP6*	rs201674756	C/T
chr7	144096938	*NOBOX*	NA	G/A	chr17	74869016	*MGAT5B*	NA	G/A
chr7	148801869	*ZNF425*	NA	C/G	chr18	72913819	*ZADH2*	rs191356988	A/G
chr7	2689594	*TTYH3*	NA	G/T	chr18	44584631	*KATNAL2*	NA	C/T
chr7	2962827	*CARD11*	rs3735133	G/A	chr19	50028070	*FCGRT*	rs374439544	C/T
chr8	8748876	*MFHAS1*	rs201875377	C/A	chr19	54743747	*LILRA6*	rs10403230	C/G
chr8	17928855	*ASAH1*	rs11538152	G/A	chr19	4504673	*PLIN4*	rs201143997	G/A
chr8	21766971	*DOK2*	rs202013016	G/A	chr19	15730502	*CYP4F8*	rs61746468	C/T
chr8	42711517	*RNF170*	rs147488061	T/C	chr19	15839677	*OR10H2*	NA	T/C
chr8	107691450	*OXR1*	rs200863692	A/G	chr19	18119274	*ARRDC2*	NA	G/A
chr8	146067346	*ZNF7*	NA	A/G	chr19	22846981	*ZNF492*	NA	A/C
chr8	52733228	*PCMTD1*	rs73592211	G/A	chr19	40743901	*AKT2*	NA	C/T
chr8	70541824	*SULF1*	NA	C/T	chr19	43420636	*PSG6*	rs370759098	G/A
chr9	2719083	*KCNV2*	rs143382624	G/C	chr19	58370766	*ZNF587*	rs77577775	G/A
chr9	18776971	*ADAMTSL1*	rs117558542	G/A	chr20	31685424	*BPIFB4*	NA	T/C
chr9	19345978	*DENND4C*	rs145052586	G/A	chr21	33690064	*URB1*	rs145519835	C/T
chr9	84226764	*TLE1*	rs141959893	C/T	chr21	37584306	*DOPEY2*	rs117132686	C/A
chr9	139750000	*MAMDC4*	rs200545888	T/C	chr21	19666690	*TMPRSS15*	NA	C/T
chr9	131670227	*LRRC8A*	NA	C/T	chr22	22673302	*IGLV5-52*	NA	C/T
chr10	25314128	*THNSL1*	rs78131600	C/T	chr22	20127408	*ZDHHC8*	rs200408305	A/G
chr10	63810739	*ARID5B*	rs201704836	G/A	chr22	46725974	*GTSE1*	rs188655025	C/G
chr10	128192832	*C10orf90*	NA	C/T	chrX	2833605	*ARSD*	rs111939179	C/T

SNP, single nucleotide polymorphism; NA, not available.

**Figure 4:**
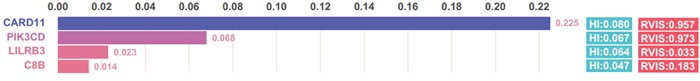
Gene prediction scores of the four genes and residual variation intolerance score of the genes

We used OMIM database and GeneCards Database to further interpret these genes and found that 4 genes among the 162 genes might have relationship with the predisposition and/or oncogenesis of AD (Figure 4). To find the candidate mutations of atopic cataracts, we searched for the genes only in this patient without in his parents. We found 10 genes mutated in this patient only without in his parents (Table 2, Supplementary Table 2). Intriguingly, we compared these genes in this special patient with the patients those had been diagnosed with cataracts and had genes mutation, so as to discuss whether these 10 genes are belonging to this special kind of disease. After analyzing the available evidence, we found no data that may suggest these genes have been reported to correlate with cataracts. It is possible that these genes may uniquely belong to AD complicated with cataracts.

**Table 2:** List of 10 genes mutated in the patient without in his parents by whole exome sequencing

Chromosome	Position	Gene	SNP	
chr12	11183066	*TAS2R31*	rs138895028	A/T
chr15	22473171	*IGHV4OR15-8*	NA	A/G
chr17	16068287	*NCOR1*	rs201932638	A/T
chr19	33490566	*RHPN2*	rs74582927	T/C
chr1	16890607	*NBPF1*	rs200783506	G/A
chr22	22730788	*IGLV5-45*	NA	G/A
chr22	22730800	*IGLV5-45*	rs114116194	A/C
chr2	90249202	*IGKV1D-43*	NA	G/A
chr2	90249205	*IGKV1D-43*	NA	A/C
chr5	140594470	*PCDHB13*	rs17844610	G/A
chr7	142149078	*TRBV5-5*	NA	T/G
chr7	142149017	*TRBV5-5*	NA	G/C
chr7	142149029	*TRBV5-5*	NA	T/G
chr7	142149030	*TRBV5-5*	NA	C/G
chr7	142149058	*TRBV5-5*	NA	T/A
chr7	142149059	*TRBV5-5*	NA	T/C
chr7	142149060	*TRBV5-5*	NA	T/C
chr7	142149066	*TRBV5-5*	NA	A/C
chr7	142149071	*TRBV5-5*	NA	T/C
chr7	142149072	*TRBV5-5*	NA	G/A
chr7	142149075	*TRBV5-5*	NA	A/G
chr7	142149086	*TRBV5-5*	NA	G/A
chr7	142149092	*TRBV5-5*	NA	T/A
chr7	142149101	*TRBV5-5*	rs199978351	A/G
chr9	33385750	*AQP7*	rs114484742	C/T

SNP, single nucleotide polymorphism; NA, not available.

## DISCUSSION

Here, we presented a rare case of AD with cataract, and familial analysis by whole exome sequencing suggested that the pathogenesis of AD was related to genetic factors. Atopic cataract was firstly described in detail in 1936, where the author demonstrated the association of juvenile cataract with AD in 10 out of 101 AD patients, the mean age was 22 year-old, similar to our findings [5]. From 1940 to 1953, an ophthalmological check in 1,158 AD patients showed typical atopic cataract in 136 patients (11.7%) including 79 cases of visual disturbance [6]. To date, literatures describing cataracts in AD are mainly from Asian populations, including the Japanese population reporting the incidence of atopic cataracts around 10-15% [7], Filipino population [8] and Singapore population without Chinese population. Based on this, it seems that a greater interest may exist in Asians, or the prevalence and significance of this disease is greater in these populations. We firstly reported cataracts in a Chinese patient with AD with cataract. Interestingly, his father and his grandma are also AD patients.

It is known that cataract may develop as a result of aging, metabolic disorders, trauma, or heredity. Location of the cataract in the lens regulates visual acuity. There are two types of cataracts in AD patients in subcapsular region, anterior subcapsular cataracts (ASCs) and posterior subcapsular cataracts (PSCs). The literatures about ASCs or PSCs development in AD patients are inconsistent. Disease onset of ASCs is typically rapid, shieldlike bilateral visual impairment [4, 9], therefore, presentation of ASCs seems to be the “classic” cataract because ASCs in the absence of AD is not common [9]. On the contrary, some investigations showed that PSCs may be more common in AD patients [9–12]. In a 29 year-old male, AD presented with bilateral ASCs [13]. Histopathologic analysis of the ASCs tissues indicated a fibrous and amorphous mass, most likely extracellular matrix owing to the presence of irregularly arranged bundled strands of fibrils, typical of collagen. Lens epithelial cells (LECs) at the plaque were densely packed and myofibroblast-like and immunoreactive for alpha-smooth muscle actin. Similarly, a 6 year-old African American girl presented with an uncontrolled flare of AD, and her medical history was significant for asthma and allergic rhinitis with a family history of AD [14]. This was in agreement with our study that the male patient's father and grandmother were also AD. Our results showed that ASCs and PSCs were both existed in the left and right lens of the patient (Figure 2).

Although the pathogenesis of AD complicated with cataract has not been clearly elucidated, severe lesions of AD located over the face may be a critical factor in the development of atopic cataracts. In addition, AD complicated with cataracts may correlate with prolonged usage of corticosteroids and repetitive periorbital scratching [11]. Physical examination of the present patient showed a scratch on the face, neck, trunk, and four extremities, especially on the face and neck, suggesting that AD complicated with cataract in this patient may correlate with scratching. However, several studies reported that the presence of cataracts (both ASCs and PSCs) were not correlated with the disease onset, severity, or duration of AD [15, 16], and the clinical features of AD patients who developed cataracts were similar to the patients who did not have. It is notable that cataract was seen in some patients with only mild facial involvement [16, 17]. On the other hand, systemic corticosteroids are known to cause ocular complications. It is reported that incidence of cataract is dose and treatment duration dependent, where the patients received the equivalent of prednisone, 10 to 15 mg/d for at least 1 year displayed the greatest risk [18]. However, Niwa, et al discussed the incidence of cataract among 3 groups of AD patients [11]. The patients were treated with topical corticosteroids, or treated with both topical and systemic corticosteroids, or corticosteroid-naive patients, respectively. The authors found no difference among these groups. Interestingly, there are 37 patients developed cataract, by which 86% showed posterior cataract [11]. This finding was similar to our study, where the patient had no history of corticosteroids. Tatham, et al reported two boys about 10 year-old diagnosed with widespread AD of the face, neck, trunk and limbs. After diagnosis and treatment with topical steroids for 2 years, both of them complained of gradual onset of blurred vision in both eyes, ophtalmic testing found PSCs in these patients, suggesting that AD and topical corticosteroids may be associated with cataracts in children [19]. Together, whether usage of corticosteroids and scratching may be susceptible factors to AD complicated with cataract is still needed to be clarified in the future with large scales of patients.

Genetic epidemiologic studies on monozygotic twins [20], and genetic association studies indicated a genetic susceptibility for AD [21]. In the present study, four genes including *CORIN*, *CARD11*, *MMP2*, *DNASE1L*, which were previously reported to be risk factors for AD [22–25], were also mutated in this patient and his father. *CARD11* encodes CARMA1, an essential scaffold protein for lymphocyte activation via T cell receptor and B cell receptor signaling [26]. CARMA1 plays important roles in T cell differentiation, regulation of JunB, GATA3 and the subsequent generation of Th2 cell specific cytokines [27]. Mice that are homozygous for the mutation affecting CARMA1 showed gradual development of AD with high level of serum IgE [28]. Li, et al [29] showed that chronic loss of epidermal caspase-8 recapitulates many aspects of AD, such as a spongiotic phenotype whereby intercellular adhesion between epidermal keratinocytes is disrupted, adversely affecting tissue architecture and function. However, subcutaneous injection of matrix metalloproteinase-2 (MMP2) inhibitor strongly down-regulated the intercellular space found in the suprabasal layers of the epidermis [29]. Suppression of MMP2 also restored full length E-cadherin to normal levels and significantly decreased the amount of the cleaved E-cadherin C-terminal fragments product. Transepidermal water loss through the epidermis from caspase-8 conditional knockout mice treated with the MMP2 inhibitor was strongly reduced relative to controls, suggesting that suppression of MMP2 is able to abrogate the effect of caspase-8 knockout induced AD. In a whole exome sequencing study of early-onset AD from a Korean population, Heo, et al discussed family-specific candidate genetic variants from three separate families, and validated the possible genes in 112 AD patients and 61 controls. Results showed that three variants of the *COL6A6* gene appeared in all three families and were in close proximity to AD related loci on chromosome 3q21 [30]. The homozygous frequency for the rs16830494 minor allele (AA) and the rs59021909 (TT) allele and the rs200963433 heterozygous (CT) frequency were all higher in AD cases compared to controls, suggesting that COL6A6 variants may be risk factors for AD.

Matsuda, et al [7] discovered that -56 T allele in the *IFNGR1* promoter was significantly associated with an increased risk of ocular AD, especially of atopic cataracts. In our study, the whole exome sequencing revealed the -56 CT genotype in both the patient and his father, which contained -56 T allele, whereas his mother harbored -56 CC genotype. The *IFNGR1* gene promoter construct that contained the -56 T allele showed higher transcriptional activity in LECs than did the construct with the -56 C allele after stimulation with IFN-γ, and there was higher IFNGR1 expression in the LECs in atopic than in senile cataracts [7], indicating that the -56 T allele in the *IFNGR1* promoter leads to elevated *IFNGR1* transcriptional activity and represents a genetic risk factor for atopic cataracts. Hori, et al [25] investigated the role of PAI-1, IFN-γ downstream molecule in the pathogenesis of atopic cataracts. They found that the IFN-γ, PAI-1 and TGF-β1 were involved in the pathophysiology of atopic cataracts.

According to the OMIM database and GeneCards Database, we found 4 genes including *CARD11, PIK3CD, LILRB3, C8B*, may correlate with the pathogenesis of AD. Among them, *CARD11* had been reported to have relation with AD [24]. Phenolyzer were used to examine the association of these candidate genes with AD and we found that *PIK3CD*, *LILRB3*, *C8B* were in the same biosystem with *CARD11* in the record of NCBI's Biosystem. According to the result of residual variation intolerance score (RVIS) [31], *CARD11* had a RVIS score of-1.39 and a percentile of 4.33%, showing that it was amongst the 4.33% most intolerant of human gene (FDR = 1.87×10^-6^), and *PIK3CD*, the 2.72% most intolerant human gene (FDR = 8.11×10^-6^), had a score of -1.66, while *LILRB3* and *C8B* with positive scores had more common functional variation. The normalized RVIS of *CARD11* and *PIK3CD* was approximated to 1, indicating that these two genes were considered as “intolerant”. *PIK3CD* had a HI score of 0.607 [32], suggesting that haploinsufficiency of the *PIK3CD* gene may associate with the pathogenesis of AD (Figure 4).

In conclusion, this is the first report of familial AD with cataracts, and the family-based whole exome sequencing found that 162 genes were both mutated in the young patient and his father, while 10 genes were only mutated in the young patient of AD complicated with cataracts. Further studies with large scale need to discuss the functional role of these genes in AD, especially in AD complicated with cataracts.

## MATERIALS AND METHODS

### Subjects

There was a 19 year-old young male with AD accompanied with cataracts. His father was AD patient, while his mother was not AD or cataracts patient. All of them were recruited in this study. The grandma was also AD, because of impossibility, the grandma was not included. Patients were collected from the Department of Dermatology of the West China Hospital Sichuan University. AD patients met the diagnostic criteria of Hanifin and Rajka [33]. Data about demographic and clinical features were collected from hospital records or by questionnaire and reviewed by experienced physicians. All subjects gave their written consent to participate before study. This study was approved by the ethics committee of the Sichuan University.

### DNA extraction and whole exome sequencing

EDTA anti-coagulated venous blood (10ml) was collected from the young male and his parents. The genomic DNA was extracted using the TIANamp Genomic DNA Kit (Tiangen Biotech, Beijing, China) following the manufacturer's protocol. Whole exome enrichment was performed using *Agilent SureSelect Human All Exon* Kit *50M* (*Agilent* Technologies, Santa Clara, CA, USA) and sequenced with the Illumina HiSeq 4000 System (HiSeq® 3000/4000 SBS Kit).

### Sequence alignment, variant calling, and annotation

The sequenced reads were aligned to the hg19 human reference genome sequence using BWA aln and BWA sampe, and removed PCR duplicates with PICARD. Variations were called by GATK HaplotypeCaller with default parameters, after calling genotyping were jointed together by GATK CombineGVCFs/GenotypeGVCFs. Variants were retained considering reads depth DP>= 8, MQ >=20. Beyond that, variants were annotated by ANNOVAR, filtered by position (non-synonymous or gain/loss of stops), VAF < 0.005 (1000 genome project (2012) and HAPMAP), potential damaging effect (variants that were predicted as damaging variants by at least 2 databases, including SIFT, PolyPhen2 HDIV, PolyPhen2 HVAR, LRT, MutationTaste, MutationAssessor, FATHMM, GERP++, PhyloP and SiPhy).

## SUPPLEMENTARY MATERIALS TABLES






