# Supplementary material for: Family-based whole exome sequencing of atopic dermatitis complicated with cataracts
Source: Oncotarget. 2017 Jul 31;8(35):59446–54. doi: 10.18632/oncotarget.19739 (PMC5601745; doi:10.18632/oncotarget.19739)
Supplement: Supplementary file 1 [file oncotarget-08-59446-s001.pdf]

Family-based whole exome sequencing of atopic dermatitis complicated with cataracts

SUPPLEMENTARY MATERIALS

Supplementary Table 1: Information about 162 genes both mutated in the patient and his father by whole exome sequencing

See Supplementary File 1

Supplementary Table 2: Information about 10 genes mutated in the patient without in his parents by whole exome sequencing

See Supplementary File 2
